# Body image, body dissatisfaction and weight status in south asian children: a cross-sectional study

**DOI:** 10.1186/1471-2458-11-21

**Published:** 2011-01-09

**Authors:** Miranda J Pallan, Lucinda C Hiam, Joan L Duda, Peymane Adab

**Affiliations:** 1 Unit of Public Health, Epidemiology & Biostatistics, University of Birmingham, Edgbaston, Birmingham, B15 2TT, UK; 2 University Hospitals Birmingham NHS Foundation Trust, Edgbaston, Birmingham, B15 2TH, UK; 3 School of Sport and Exercise Sciences, University of Birmingham, Edgbaston, Birmingham, B15 2TT, UK

## Abstract

**Background:**

Childhood obesity is a continuing problem in the UK and South Asian children represent a group that are particularly vulnerable to its health consequences. The relationship between body dissatisfaction and obesity is well documented in older children and adults, but is less clear in young children, particularly South Asians. A better understanding of this relationship in young South Asian children will inform the design and delivery of obesity intervention programmes. The aim of this study is to describe body image size perception and dissatisfaction, and their relationship to weight status in primary school aged UK South Asian children.

**Methods:**

Objective measures of height and weight were undertaken on 574 predominantly South Asian children aged 5-7 (296 boys and 278 girls). BMI z-scores, and weight status (underweight, healthy weight, overweight or obese) were calculated based on the UK 1990 BMI reference charts. Figure rating scales were used to assess perceived body image size (asking children to identify their perceived body size) and dissatisfaction (difference between perceived current and ideal body size). The relationship between these and weight status were examined using multivariate analyses.

**Results:**

Perceived body image size was positively associated with weight status (partial regression coefficient for overweight/obese vs. non-overweight/obese was 0.63 (95% CI 0.26-0.99) and for BMI z-score was 0.21 (95% CI 0.10-0.31), adjusted for sex, age and ethnicity). Body dissatisfaction was also associated with weight status, with overweight and obese children more likely to select thinner ideal body size than healthy weight children (adjusted partial regression coefficient for overweight/obese vs. non-overweight/obese was 1.47 (95% CI 0.99-1.96) and for BMI z-score was 0.54 (95% CI 0.40-0.67)).

**Conclusions:**

Awareness of body image size and increasing body dissatisfaction with higher weight status is established at a young age in this population. This needs to be considered when designing interventions to reduce obesity in young children, in terms of both benefits and harms.

## Background

Obesity presents major health threat around the globe. Within the UK, prevalence of overweight and obesity has escalated in the last 30 years[1], and tackling this is a government priority[2]. There is a particular focus on children, where rates of obesity are predicted to treble over the next 50 years[3] with consequent impact on adult obesity and its related health consequences[4,5]. Psychosocial factors are particularly important in childhood obesity[4,6] and need to be better understood and considered when designing prevention interventions.

Body image is a psychosocial dimension, defined by Schilder in the 1930 s as 'the picture of our own body which we form in our own mind'[7]. It is clear, however, from emergent research in the field, that body image is a multidimensional concept, incorporating neurological, psychological and sociocultural elements, and the complexity of the construct is not reflected in Schilder's definition[8]. Body image is viewed as central to many aspects of human functioning, including emotions, thoughts, behaviours and relationships. Therefore, the effect of body image on quality of life is potentially extensive[8].

Much of the research into body image has been in the field of eating disorders. There are two types of disturbance of body image; perceptual and attitudinal. Perceptual disturbance relates to over or underestimation of body size, and attitudinal disturbance involves dissatisfaction with body shape or size [9]. Perceptual and attitudinal body image distortion correlates with increased psychological distress and disordered eating[10], and body image distortion is a diagnostic criterion for both anorexia and bulimia nervosa[11]. Obesity is also recognised as part of the spectrum of conditions related to disordered eating, and with the escalation of the obesity problem, its relationship to body image and dissatisfaction has become a focus of research[10,12]. A consistent relationship between increasing weight status and body dissatisfaction in older children has been observed in many different cultural communities[13-23]. In younger children however, the association is less clear[24]. Relatively little is known about body image development in children, and most studies in this area involve small, predominantly white samples. A negative attitude towards a fat body shape has been observed in children as young as 4[25], although the emergence of actual body dissatisfaction appears to be sometime after this, becoming more pronounced in middle childhood and early adolescence[26,27]. In a US study looking at weight concerns and body dissatisfaction in 5 year old girls, Davison et al. found an association between body dissatisfaction and weight status, but overall, body dissatisfaction was relatively infrequent in this age group[24]. In a longitudinal study of the same cohort no significant differences in weight concern and body dissatisfaction were found when comparing normal weight and overweight at age 5, but by age 9, the differences were highly significant[28,29]. Significant longitudinal associations between weight status in childhood and later body dissatisfaction have also been found. Several studies report that overweight or obesity in childhood or adolescence predicts body dissatisfaction at a later stage in both sexes[18,21,29-32]. Body dissatisfaction in turn is linked to later weight status, and associated behaviours. Body dissatisfaction in adolescence has been associated with excess weight gain in the following years[33,34], and with later unhealthy behaviours such as binge eating, unhealthy dieting, decreased physical activity and unhealthy weight control behaviours[35]. In addition, early childhood body dissatisfaction in girls has been found to be associated with later maladaptive eating attitudes and dieting[28].

One problem in examining body image and body dissatisfaction in children and in comparing different studies is the lack of consistency in measurement instruments and the limited range of validated measures. In general, there are two types of measures; figure rating scales and questionnaires, each with their own merits and limitations[26,27]. The former consist of a series of figure drawings ranging from very thin to obese, and participants can indicate the figure they most think they resemble. These are easy to administer to children, with high test-retest reliability in those age 6 and above[36,37]. The validity of these instruments in younger children is unknown, although despite some biases[38] significant correlation between selected figures and actual body size (measured by BMI) has been found in children as young as 7. Questionnaire measures range from simple questions such as 'how satisfied are you with your appearance[27] to the Eating Disorders Inventory Body Dissatisfaction Scale (which assesses beliefs around various body parts and fatness)[39] and the Body Esteem Scale (which assesses children's attitudes and feelings about their bodies)[40]. The validity and reliability of these instruments in children younger than 8 is unknown[26,27].

Sex differences in body dissatisfaction and its relationship to weight status have been found among older children[26]. Body dissatisfaction and a desire to be thinner are equally prevalent among overweight boys and girls. However underweight boys attach importance to having big muscles[20,21,41] and are therefore more likely to desire a larger body size than underweight girls[42].

Variation in body dissatisfaction related to weight status by ethnic group has also been studied. Unsurprisingly, the influence of ethnicity on body dissatisfaction is complex and interacts with other factors, such as societal, media and peer influences[14,22,23,26,43]. Some patterns have nevertheless emerged. For example, body dissatisfaction among overweight African-American children is less marked than among their white counterparts[26,43,44], although this is not consistent across all settings[22,45]. On the other hand, among Native American Indians, with high prevalence of obesity and diabetes[46], body dissatisfaction was positively associated with weight status among children[14,18]. This contrasts with previous beliefs that obesity is culturally acceptable in these populations.

Little research into body dissatisfaction and its association with weight status in UK South Asian children has been undertaken. This group represent a particular target for obesity prevention, as the incidence of obesity-related diseases are greater than among the general UK population[47,48]. A better understanding of ethnic and cultural norms related to obesity among this heterogeneous community is essential for planning interventions. One study which included South Asians reported a strong association between body fatness and body dissatisfaction among adolescents[19], but it is not known whether this association holds true for younger children.

In summary, currently there is insufficient understanding of when the relationship between body dissatisfaction and weight status develops in children and what the correlates of such a relationship are, particularly in relation to ethnicity. In this study we use baseline data from a childhood obesity prevention study to explore body image size and dissatisfaction and its relationship to weight status in a UK sample of South Asian boys and girls aged 5 to 7 years. This information is important for informing approaches to obesity prevention and management in children from these ethnic groups.

## Methods

### Participants

Participants were part of the Birmingham Healthy Eating and Active Lifestyle for Children Study (BEACHeS), which aimed to develop and pilot a childhood obesity prevention intervention, focusing on South Asians. For the main study, 574 children (of a possible 1090; 53%) in years 1 and 2 (age 5-7) from eight primary schools in Birmingham, a major UK city, participated. Schools with predominantly South Asian children were selected. Written consent was obtained from parents of the participating children, and verbal assent obtained from the participating children. The study was reviewed and approved by the East Birmingham Local Research Ethics Committee. Data on ethnicity were obtained from school records, based on parental report. The mean study sample age was 6.5 years (SD = 0.6) with equal gender split. The ethnic breakdown of participants was South Asian: Pakistani 66.9%, Bangladeshi 14.3%, Indian 4.7%; Black 7.8%; White 2.3%; other 4.0%. Each child's home postcode was used to assign a Townsend deprivation score based on the 2001 census and scores were compared to the ranking of scores assigned to postcode areas within the West Midlands Government Office Region. Ninety-four percent of children had postcodes that were within the most deprived decile for the region.

### Measurement of weight status

Trained researchers undertook height and weight measurements using standardised instruments and procedures. Height was measured to the nearest 0.1 centimetres using a Leicester height measure. Weight was measured in light clothing and no shoes to the nearest 0.1 kg using a Tanita TBF 300 MA body composition analyser. Body Mass Index was calculated by the formula; weight (kg)/height (m)^2 ^and standard deviation scores (BMI z-scores) derived using the age and sex specific UK National 1990 BMI percentiles reference data[49]. Children were also categorised as underweight, healthy weight, overweight and obese, according to the UK 1990 reference data, using the 2^nd^, 85^th ^and 95^th ^percentiles to define these categories. These definitions are in line with national childhood obesity prevalence monitoring programmes (such as the National Child Measurement Programme), and the 95^th ^percentile definition for obesity has been shown to have a high sensitivity and specificity[50].

### Measurement of body image

A figure rating scale, developed by Collins[36] and adapted by Rand and Resnick[51] was used to assess body image. The 3 day test-retest reliability coefficients of the instrument developed by Collins are 0.71 for perceived self image and 0.59 for ideal self image for children age 6 to 9. The correlation between perceived self image size and BMI was 0.37 in the same age group[36]. Gender and skin colour specific drawings consisted of 9 prepubescent figures ranging from very underweight (value = 1) to very overweight (value = 9). Children were asked; "Which picture looks the most like you look?", and "Which picture shows the way you want to look?" to assess 'self' and 'ideal self' respectively (scores ranging from 1 to 9 for each). A body dissatisfaction (BDS) score was derived by subtracting the 'ideal self' from the 'self' score, (scores ranging from -8 to +8). Thus negative or positive scores indicated the child perceived him/herself as thinner or more overweight than ideal respectively, whilst a zero score indicated they were satisfied.

### Procedure

Data collection took place from December 2006 to May 2007. On the day of data collection, small groups of children (4) were taken from class to a separate room, and rotated around four separate data collection stations, consisting of either physical measures, or psychosocial instruments. Children were with trained researchers on a one-to-one basis at each station. Researchers followed standard operating procedures for both physical and psychosocial measures. In the case of the latter, there were clear guidelines to ensure children understood the questions that they were being asked, including repetition of the question and checking of their understanding.

### Analysis

Analysis was undertaken using the STATA (version 10) statistical package. The outcome variables explored were perception of self, perception of ideal self, and body dissatisfaction scores. For bivariate categorical comparisons, chi squared tests were performed. Multiple regression analyses were performed for each outcome, with weight status, sex, age and ethnicity included as independent variables. Two alternative variables were used for weight status; BMI z-score and binary weight category (not overweight/obese versus overweight/obese). Ethnicity was categorised into 4 groups; Pakistani, Indian, Bangladeshi and Other.

## Results

Data on age, gender, ethnicity and weight status were obtained for all 574 children, and 571 children completed the body image assessment.

### Weight status

The mean BMI z-score for the sample was 0.03 (SD 1.38). One fifth of participants were either overweight or obese, with girls significantly more likely to be both overweight and obese (Table 1). There were no significant differences across different ethnic or age groups.

**Table 1:** Weight status of study sample by sex, age and ethnicity

	Number (%) underweight	Number (%) healthy weight	Number (%) overweight	Number (%) obese
Total	15 (2.6)	440 (77.2)	42 (7.4)	73 (12.8)
Sex*				
Boys	5 (1.7)	242 (82.0)	17 (5.8)	31 (10.5)
Girls	10 (3.7)	198 (72.0)	25 (9.1)	42 (15.3)
Age†				
Age 5	2 (1.4)	107 (75.4)	12 (8.5)	21 (14.8)
Age 6	8 (2.7)	230 (77.7)	20 (6.8)	38 (12.8)
Age 7	5 (3.8)	103 (78.0)	10 (7.6)	14 (10.6)
Ethnicity‡				
Bangladeshi	0 (0.0)	60 (74.1)	8 (9.9)	13 (16.1)
Indian	0 (0.0)	24 (88.9)	0 (0.0)	3 (11.1)
Pakistani	14 (3.7)	297 (77.8)	23 (6.0)	48 (12.6)
Other	1 (1.3)	59 (73.8)	11 (13.8)	9 (11.3)

* χ^2 ^= 8.56 (df = 3), p = 0.04

† χ^2 ^= 2.89 (df = 6), p = 0.82

‡ χ^2 ^= 14.63 (df = 9), p = 0.10

### Perception of body image (self)

The median self image score for the total sample was 4 (IQR = 2-5). The distribution of scores (Table 2) was similar across sexes, age, and ethnic groups, except the Indian sub-group, which had a lower median score (M = 3, IQR 1-4). The obese group had a higher median score than other categories (M = 5, IQR = 4-5)

**Table 2:** Distribution of perceived self and ideal self scores by sex

Figure rating score	Number (%) choosing score for 'self' image		Number (%) choosing score for 'ideal self' image	
	**Boys**	**Girls**	**Boys**	**Girls**

1 (thinnest)	42 (14.2)	63 (22.7)	55 (18.7)	78 (28.2)
2	23 (7.8)	32 (11.6)	25 (8.5)	42 (15.2)
3	34 (11.5)	39 (14.1)	43 (14.6)	45 (16.3)
4	73 (24.8)	54 (19.5)	70 (23.8)	54 (19.5)
5	86 (29.2)	67 (24.2)	55 (18.7)	39 (14.1)
6	18 (6.1)	18 (6.5)	24 (8.2)	14 (5.1)
7	12 (4.1)	0 (0.0)	6 (2.0)	1 (0.4)
8	1 (0.3)	0 (0.0)	2 (0.7)	3 (1.1)
9 (fattest)	6 (2.0)	4 (1.4)	14 (4.8)	1 (0.4)

Multiple regression models indicated that lower BMI z-score (or not being overweight/obese) was significantly associated with lower "self" image size perception scores (thinner self image) (tables 3 and 4). Ethnicity (Indian subgroup) and sex (being female) were also significantly associated with thinner self perception scores. The magnitudes of the associations were small. Subgroup analysis by sex found that the association between weight status and self perception image size was only significant for girls.

**Table 3:** Linear regression models to examine predictors for perceived self, ideal self and body dissatisfaction scores (BMI z-score as weight predictor variable)

Variable	Perceived self (Adjusted R^2 ^= 0.06, F = 6.16 (df = 6), p < 0.0001)	Perceived ideal self (Adjusted R^2 ^= 0.10, F = 11.45 (df = 6), p < 0.0001)	Body dissatisfaction (Adjusted R^2 ^= 0.09, F = 10.88 (df = 6), p < 0.0001)
	**β (95% CI)**	**β (95% CI)**	**β (95% CI)**

↑ BMI z-score	0.21*** (0.10-0.31)	-0.33*** (-0.44--0.22)	0.54*** (0.40-0.67)
Female†	-0.60*** (-0.89--0.31)	-0.78*** (-1.0--0.47)	0.18 (-0.20-0.56)
↑ age (months)	0.004 (-0.02-0.02)	-0.03* (-0.05--0.004)	0.03* (0.003-0.06)
Indian‡	-0.90* (-1.67--0.13)	-0.43 (-1.24-0.37)	-0.47 (-1.48-0.54)
Pakistani	-0.03 (-0.4-0.39)	0.03 (-0.41-0.48)	-0.07 (-0.63-0.49)
Other	-0.09 (-0.63-0.46)	0.28 (-0.28-0.85)	-0.37 (-1.08-0.34)

Reference is: † male, ‡ Bangladeshi

*p < 0.05, **p < 0.01, ***p < 0.001

**Table 4:** Linear regression models to examine perceived self, ideal self and body dissatisfaction scores in relation to being overweight/obese vs. not overweight/obese

Variable	Perceived self (Adjusted R^2 ^= 0.05, F = 5.56 (df = 6), p < 0.0001)	Perceived ideal self (Adjusted R^2 ^= 0.08, F = 8.71 (df = 6), p < 0.0001)	Body dissatisfaction (Adjusted R^2 ^= 0.06, F = 7.14 (df = 6), p < 0.0001)
	**β (95% CI)**	**β (95% CI)**	**β (95% CI)**

Overweight/ obese†	0.63** (0.26-0.99)	-0.85*** (-1.23--0.46)	1.47*** (0.99-1.96)
Female‡	-0.63*** (-0.92--0.33)	-0.75*** (-1.06--0.44)	0.13 (-0.26-0.52)
↑ age (months)	0.003 (-0.02-0.02)	-0.02* (-0.05--0.003)	0.03 (<0.001-0.06)
Indian§	-0.93* (-1.70--0.16)	-0.37 (-1.19-0.44)	-0.55 (-1.58-0.47)
Pakistani	-0.07 (-0.50-0.35)	0.11 (-0.34-0.56)	-0.19 (-0.75-0.38)
Other	-0.12 (-0.67-0.42)	0.34 (-0.23-0.92)	-0.47 (-1.19-0.26)

Reference is: † not overweight/obese, ‡ male, § Bangladeshi

*p < 0.05, **p < 0.01, ***p < 0.001

### Perception of ideal self

The median score for "ideal self" was lower than that for "self" image size (M = 3, IQR = 2-5). Girls had a lower median score than boys. Distribution of scores is shown in table 2 and did not differ greatly across age and ethnic groups, although Indians had the lowest median score. In contrast with the self image scores, the obese category had the lowest median score for ideal self image (M = 2, IQR = 1-4).

Multiple regression models showed that adjusted for other factors, increasing BMI z-score (or being overweight/obese) was significantly associated with lower ideal self scores (thinner shapes) (tables 3 and 4), and on stratified analysis, this finding was consistent in both sexes. Increasing age and female sex were also significantly associated with lower ideal self scores. Again, the magnitudes of the associations were small.

### Body dissatisfaction

The mean body dissatisfaction score for the study sample was 0.26 (SD 2.41). Mean scores for the different weight categories were 0.33 (SD 2.26), -0.05 (SD 2.35), 0.71 (SD 2.12) and 1.86 (SD 2.38) for underweight, healthy weight, overweight and obese respectively. Eighteen percent were satisfied with their body size (BDS score = 0), 37.3% perceived themselves as too thin (BDS score < 0), and 44.7% as too fat (BDS > 0, table 5). Mean scores across weight categories differed significantly (F = 14.51, p < 0.001), with the overweight and obese categories having higher dissatisfaction scores than the healthy and underweight categories.

**Table 5:** Body dissatisfaction by sex, age, ethnicity and weight status

	Number (%) perceives self as too thin (BDS score < 0)	Number (%) satisfied (BDS score = 0)	Number (%) perceives self as too fat (BDS score > 0)
Total	212 (37.3)	102 (18.0)	254 (44.7)
Sex*			
Boys	116 (39.5)	57 (19.4)	121 (41.2)
Girls	96 (34.7)	48 (17.3)	133 (48.0)
Age†			
Age 5	59 (41.3)	30 (21.0)	54 (37.8)
Age 6	111 (37.5)	46 (15.5)	139 (47.0)
Age 7	42 (31.8)	29 (22.0)	61 (46.2)
Ethnicity‡			
Bangladeshi	25 (30.9)	18 (22.2)	38 (46.9)
Indian	15 (55.6)	1 (3.7)	11 (40.7)
Pakistani	140 (36.7)	77 (20.2)	165 (43.2)
Other	39.5 (32)	11.1 (9)	49.4 (40)
Weight category§			
Underweight	4 (26.7)	4 (26.7)	7 (46.7)
Healthy weight	186 (42.5)	80 (18.3)	172 (39.3)
Overweight	11 (26.2)	9 (21.4)	22 (52.4)
Obese	11 (15.1)	9 (12.3)	53 (72.6)

* χ^2 ^= 2.72 (df = 2), p = 0.26

† χ^2 ^= 6.37 (df = 4), p = 0.17

‡ χ^2 ^= 11.04 (df = 6), p = 0.09

§ χ^2 ^= 33.03 (df = 6), p < 0.001

Multiple regression analyses indicated that adjusted for other factors, increasing body dissatisfaction score was associated with increasing BMI z-score (or overweight/obese) and increasing age. There was no significant association with gender or ethnic subgroup (tables 3 and 4).

## Discussion

We found a high rate of body dissatisfaction (over 80%) and a significant relationship between objectively measured weight status and body image perception and body dissatisfaction in UK South Asian children as young as 5. The findings indicate that even at this age, children have some self-awareness about their weight status and a concept of societal norms. The finding that self image perception is associated with weight status only in girls implies that they may be more aware of their body shape than boys at this age, although overweight and obese boys and girls appear to suffer similar levels of body dissatisfaction.

The association between increasing weight status and body dissatisfaction found in this study has been little explored in such young children in the past. Previous studies based on small samples (n < 200) of mostly white girls in Pennsylvania have shown an association that becomes clearer with increasing age, such that by the age of 8, the relationship between increasing weight and body dissatisfaction is firmly established[24,28,29]. We also found that the association was stronger with increasing age, but our findings based on a large, mixed sex sample suggests that body dissatisfaction with increasing weight status is established even in the youngest age group and is seen in both boys and girls.

Other studies have reported sex differences in body dissatisfaction, although these were generally observed among children over the age of 8[26,32,52]. Our findings suggest that sex differences in body dissatisfaction are not apparent in early childhood in this population. It may be that such differences will become apparent in the adolescent period, or that differences are less pronounced in this ethnic group. A Malaysian study of body dissatisfaction in adolescents found a sex difference in Chinese, but not in Malay or Indian adolescents[53].

Weak but significant associations between female sex and lower scores for perceived self and perceived ideal self were found, indicating that on average, the perceived norms for body size are smaller for girls compared to boys. The sex difference in size for ideal self is coherent with previous findings that boys generally prefer larger body size and attach importance to being muscular, along with concerns about weight[20,21,41,52].

Marked differences in cultural norms, media influences, religious and dietary practices suggest that differences between Indian, Pakistani and Bangladeshi subgroups would be expected. Studies looking at different ethnic populations of older children have reported differences in body image and body dissatisfaction, although the findings have not always supported prior assumptions about the ethnic groups under study[14,18,19,22,53]. In this study, we found few ethnic subgroup differences in the three outcomes. Indian children tended to have lower weight status and smaller ideal body image size compared to the other South Asian groups. However, the small number of subjects in Indian subgroup (n = 27), makes interpretation difficult, and suggests further exploration is needed.

The perceived cultural norms of the UK South Asian population that overweight is acceptable, even desirable[54], may lead one to hypothesise that there would be no association between weight and body dissatisfaction in these children, but this does not appear to be the case. The findings of this study demonstrate that the association exists, and this is consistent with other research. A stronger association between body fat and body dissatisfaction was found in UK South Asian adolescents, compared to black African Caribbeans and whites [19], and a further study found a desire for thinness in preadolescent British Asian and Caucasian girls to be comparable[55].

Although not the main focus of the study, an interesting finding is the large proportion of children who had BDS scores which imply that they perceive themselves as too thin (37%), when less than 3% are underweight. This merits further exploration as this finding may also have implications in terms of obesity intervention.

The findings of this study add to the existing body of knowledge on weight status and body image for a number of reasons. Firstly, this is the largest study to explore the association in such a young age group, and to include both sexes. The study has also focused on a large immigrant group in the UK that is known to be at risk of obesity and its health consequences[48]. Attempting to understand the psychosocial functioning in South Asian children in relation to obesity is crucial when developing interventions to reduce obesity in this group. This becomes especially important if one considers the potential conflicting family, community and wider societal influences on South Asian children. We have also begun to explore the differences in weight status and body image in the different South Asian subgroups, and again, it is important in obesity intervention planning that South Asians are not regarded as one homogeneous group.

This study has several limitations. The study population was predominantly South Asian and the vast majority were from households in areas of deprivation. This made it difficult to study the influences of ethnicity and socioeconomic status on body dissatisfaction and its relationship with overweight and obesity, and the study could have been improved by adding both white and black comparator groups, and having subjects from across the socioeconomic spectrum. The response rate for the study was 53%, and it is possible that the non-respondents' characteristics differed from respondents', for example, parents of more overweight children may be less likely to consent to their child participating in this study. However, the weight distribution of the sample population was similar to that reported for Birmingham as a whole, which makes such bias less likely. Another limitation is the use of the adapted Collins Figure Rating scale to assess body image. The reliability and validity of the instrument has only been demonstrated in children aged 6 or over, whereas around one fifth of the sample population were aged 5 at the time of the study. The findings therefore have to be interpreted with this in mind. In terms of assessing body dissatisfaction, it is the discrepancy between the child's perception of themselves and their perceived ideal that is of interest, and so reliability is of greater importance than how their perceived self relates to their actual body size. Finally, the derivation of body dissatisfaction in this study makes the assumption that a child is dissatisfied with their body if their chosen ideal figure differs from their perceived self. This is not necessarily the case and children may have range of body shapes that they find socially acceptable, as demonstrated by Rand and Resnick[51].

## Conclusions

The findings of this study support the idea that even at a young age children have some awareness of their body size and of some societal norms, with more overweight children experiencing higher body dissatisfaction and a desire to be thinner. This raises questions as to whether children as young as 5 are subject to societal influences such as the media[56] that portray the thin ideal. In South Asian children, matters are further complicated as these pervasive influences may create conflict with traditional family and community values. Areas for future research include an exploration of the longitudinal relationship between body image and dissatisfaction and future weight status in order to determine whether accuracy of weight perception or body dissatisfaction are potential predictors of weight change. Given the difficulties in assessing body image in young children, more work also needs to be done on developing valid and reliable measures for use in very young children.

These findings have potential implications for childhood obesity intervention. If overweight and obesity leads to higher levels of body dissatisfaction in young children, as this study suggests, all components of intervention programmes aimed at reducing obesity in younger children should take this into account, and should be designed with sensitivity towards this issue. Those developing obesity interventions that target UK South Asian children need to pay particular attention to the psychosocial functioning of these children and the possible internal conflicts they may face. With this approach, interventions will minimise potential harm to children and will be beneficial, not only in terms of reducing obesity, but also by maximising psychosocial health.

## Competing interests

The authors declare that they have no competing interests.

## Authors' contributions

MP participated in study design, fieldwork and analysis, and drafted the manuscript. LH participated in the fieldwork and analysis, and helped to draft the manuscript. JD participated in design of the study and helped draft the manuscript. PA had overall responsibility for the BEACHeS study, participated in study design, fieldwork and analysis, and helped draft the manuscript. All authors read and approved the final manuscript.

## Pre-publication history

The pre-publication history for this paper can be accessed here:

http://www.biomedcentral.com/1471-2458/11/21/prepub
